# To the Editors of the Medical and Physical Journal

**Published:** 1801-04

**Authors:** Henry Headly

**Affiliations:** Calne, Wilts


### *To the Editors of the Medical and Phyſical Journal.*

Gentlemen,

WITH the view of benefitting thoſe unfortunate females who are afflicted with ſuch deformity of the pelvis as precludes the poſſibility of their bearing full-ſized live children, I beg leave to relate one of two ſucceſsful caſes of premature delivery, firſt recommended by Dr. Denman, which I have lately been engaged in.

In the beginning of January, I was conſulted by a woman of the above deſcription, the wife of a reſpectable farmer who lives about ſeven miles from this place. She told me, that ſhe then wanted ſix weeks of her natural time of delivery, and was willing to ſubmit to any operation that would give her the chance of a live child. Finding ſhe had delayed applying to me a fortnight longer than ſhe ought to have done, I immediately ſent for the gentleman who had before attended her; and learnt that her two firſt children were feet preſentations; and the latter, thoſe of the head; that the neceſſary force uſed in extracting the præternatural preſentations deſtroyed life; and that embryotomy, in the three laſt caſes, was performed with the utmoſt difficulty, from diſtortion preventing the head ſinking into the pelvis; I alſo underſtood that he had been twice aſſiſted by Dr. L—w, a man of great and deſerved eminence in his profeſſion; and I have no doubt but the caſes were conducted with great patience and judgment.

I immediately propoſed rupturing the membranes, to which my friend not only cordially aſſented, but offered to attend the parturition. To be conciſe, I ſhall only obſerve that the os uteri was high up, inclined backward, and cloſely contracted; but by moderate preſſure, and a rotatory motion made with the finger, it was ſoon dilated, ſufficiently for me to introduce a male catheter, ſomewhat ſtraightened. This, however, not anſwering the purpoſe of rupturing the membranes, I introduced another inſtrument, a little more pointed, and diſcharged between three and four pints of water. About twenty hours after, my patient was ſeized with ſtrong labour pains, and with great difficulty delivered of a live child. The child was ſmaller than uſual, and the preſentation footling.

I am, your's, &c.

*Calne, Wilts, Mar.* 16, 1801. HENRY HEADLY.

*Prize*

